# The COVID-19 Pandemic Mental Health Questionnaire (CoPaQ): psychometric evaluation and compliance with countermeasures in psychiatric inpatients and non-clinical individuals

**DOI:** 10.1186/s12888-021-03425-6

**Published:** 2021-08-31

**Authors:** Stephanie V. Rek, Markus Bühner, Matthias A. Reinhard, Daniel Freeman, Daniel Keeser, Kristina Adorjan, Peter Falkai, Frank Padberg

**Affiliations:** 1 Department of Psychiatry and Psychotherapy, LMU University Hospital Munich, Nussbaumstraße 7, Munich, Germany; 2 International Max Planck Research School for Translational Psychiatry (IMPRS-TP), Munich, Germany; 3 Department of Psychology, LMU Munich, Munich, Germany; 4 Department of Psychiatry, University of Oxford, Oxford, UK

**Keywords:** Coronavirus, Psychosocial impact, Questionnaire validation, Factor analysis, Preventive countermeasures

## Abstract

**Background:**

The COVID-19 pandemic has greatly impacted people’s lives across a broad spectrum of psychosocial domains. We report the development and psychometric evaluation of the self-report COVID-19 Pandemic Mental Health Questionnaire (CoPaQ), which assesses COVID-19 contamination anxiety, countermeasure necessity and compliance, mental health impact, stressor impact, social media usage, interpersonal conflicts, paranoid ideations, institutional & political trust, conspiracy beliefs, and social cohesion. Further, we illustrate the questionnaire’s utility in an applied example investigating if higher SARS-Cov-2 infection rates in psychiatric patients could be explained by reduced compliance with preventive countermeasures.

**Methods:**

A group of 511 non-clinical individuals completed an initial pool of 111 CoPaQ items (Open Science Framework: https://osf.io/3evn9/) and additional scales measuring psychological distress, well-being, and paranoia to assess construct validity and lifetime mental health diagnosis for criterion validity. Factor structure was determined by exploratory factor analyses and validated by conducting confirmatory factor analysis in the accompanying longitudinal sample (*n* = 318) and an independent psychiatric inpatient sample primarily admitted for major depressive-, substance abuse-, personality-, and anxiety disorders (*n* = 113). Internal consistency was assessed by Cronbach’s Alpha and McDonald’s Omega. For the applied research example, Welch t-tests and correlational analyses were conducted.

**Results:**

Twelve out of 16 extracted subscales were retained in the final questionnaire version, which provided preliminary evidence for adequate psychometric properties in terms of factor structure, internal consistency, and construct and criterion validity. Our applied research example showed that patients exhibited greater support for COVID-19 countermeasures than non-clinical individuals. However, this requires replication in future studies.

**Conclusions:**

We demonstrate that the CoPaQ is a comprehensive and valid measure of the psychosocial impact of the pandemic and could allow to a degree to disentangle the complex psychosocial phenomena of the pandemic as exemplified by our applied analyses.

**Supplementary Information:**

The online version contains supplementary material available at 10.1186/s12888-021-03425-6.

## Introduction

The COVID-19 pandemic and related governmental restrictions and recommendations to contain the rapid spread of the coronavirus (e.g., stay-at-home orders & social distancing) have greatly changed people’s lives. Early on, news outlets and initial research have cautioned that the COVID-19 pandemic would affect and be affected by a number of key aspects of individuals’ lives. These aspects include mental health and pandemic-related stress, risk and protective factors (contamination anxiety, social media usage, interpersonal conflicts, mental health-protective behaviour), and individuals’ perception of the political handling of the crisis (conspiracy beliefs, political and institutional trust, and support of public health directives; e.g. [1–3],). We developed the COVID-19 Pandemic Mental Health Questionnaire (CoPaQ) in order to capture this wide pandemic-related psychosocial spectrum applicable for different study populations. The self-report questionnaire was published on the Open Science Framework (OSF; https://osf.io/3evn9/) in German and English early on in the pandemic in April, 2020 [4]. Since then, the instrument has received recognition from the wider research community worldwide with translations into Spanish, Croatian, Portuguese, Greek, Hungarian, Korean, Nepalese, Czech, and Romanian illustrating that the questionnaire has been well received and was applied frequently in different countries and study populations. To date, however, a psychometric validation of the CoPaQ has been lacking.

The present study aims to provide a comprehensive description and psychometric evaluation of the CoPaQ. We recruited a group of non-clinical individuals online (*n* = 511) and psychiatric inpatients from the LMU Biobank study (*n* = 113). They completed the CoPaQ and validated self-report measures on psychological distress, wellbeing, and paranoia. To determine the factor structure, selection of items, and model fit, we applied exploratory factor analyses (EFA) in our non-clinical derivation sample. We then evaluated this factor structure by applying confirmatory factor analyses (CFA) using accompanying longitudinal 10-week follow-up data (*n* = 318) and separate CFA for the psychiatric inpatient sample. Internal consistency was determined using McDonald’s Omega and Cronbach’s Alpha across samples. For selected subscales of the CoPaQ, we evaluated criterion- and construct validity.

In addition to the psychometric validation of the CoPaQ, we illustrate the questionnaire’s utility in an applied research example. Previously, two large US cohort studies have suggested that patients with a history of mental disorders have an increased risk for SARS-Cov-2 infection even when controlling for important socioeconomic and health-related factors [5, 6]. The authors suggest that one explanation for the increased risk of SARS-Cov-2 infection could be patients’ lower compliance with public health directives (hygiene measures, social distancing guidelines, and political restrictions). However, this hypothesis and potential explanatory factors have not been investigated empirically.

A number of factors have been associated with individual differences in levels of compliance in the public with governmental guidelines. Higher levels of an individual’s risk perception [7–9] as well as political and institutional trust [10] have been found to increase support for governmental regulations. Contrary to this, erroneous conspiracy beliefs about the origin of the coronavirus have been associated with reduced adherence to preventive measures [2]. Consequently, multiple plausible and partly diverging hypotheses could explain potential differences in support of governmental restrictions and regulations between psychiatric patients and non-clinical individuals. First, lower levels of trust may promote less adherent behaviours in psychiatric patients, which may be reflected in lower levels of COVID-19 institutional & political trust and higher levels of COVID-19 conspiracy beliefs and paranoid ideations, compared to non-clinical individuals. Conversely, psychiatric patients’ adherence to COVID-19 countermeasures could be greater than in non-clinical controls as patients may exhibit higher levels of COVID-19 contamination anxiety, more COVID-19 physical risk factors, and overall greater general anxiety symptoms. These fear-related characteristics could promote protective behaviours against SARS-Cov-2 infection as reflected in higher levels of COVID-19 contamination anxiety, COVID-19 physical risk factors and general anxiety symptoms, compared to non-clinical individuals. Here, psychiatric patient populations may differ in their levels of mistrust, which is most characteristic for psychotic disorders and fear, which is most characteristic for anxiety disorders. In this study, we focused on a transdiagnostic psychiatric inpatient sample from all major diagnostic categories with most prominent prevalence of major depressive-, substance abuse-, personality-, and anxiety disorders. Testing these hypotheses requires the assessment of a number of COVID-19 related psychosocial domains, which provides the ideal setting to explore the utility of the CoPaQ questionnaire in the context of our case-control sample.

## Study Part 1: Questionnaire validation

## Methods

### CoPaQ construction

The initial item pool was devised by the study team (psychiatrist and clinical psychologists) based upon clinical experience, reference to the current diagnostic classification system of Diagnostic And Statistical Manual Of Mental Disorders, fifth edition [11], reference to existing measures [12], and an extensive internet search for current media and research outputs [13–16]. Thereafter, each item was evaluated with regard to its face and content validity by independent experts (two examinees). A final construct of questions was designed, ensuring no overlap. Due attention was given to ensure that the questions were framed in simple language, and worded positively, with no ambiguity.

The first part of the questionnaire served to characterise the population under study by asking about SARS-Cov-2 infection status, COVID-19 physical health risk factors (self/others), employment status, health insurance status, life time mental health diagnosis, etc. The subsequent item pool was devised to reflect the following COVID-19-related constructs: contamination anxiety (9 items), necessity of and compliance with countermeasures (29 items), mental health symptomatology (25 items), positive coping (12 items), stressors (29 items), interpersonal conflicts (5 items), social media usage (7 items), political and institutional trust (6 items), paranoid ideations (5 items), conspiracy beliefs (7 items), and social cohesion (6 items). The time period for all the items was either relating to the present moment or the previous 2 weeks. Items were rated on a 0 (not at all) to 4 (very much) scale.

We disseminated the questionnaire to the wider research community prior to validation to facilitate its use during the rapidly unfolding events during the pandemic.

### Participants

To extract the items for the new measures of the psychosocial impact of the COVID-19 pandemic, a derivation sample of 511 participants from the general German population completed the full item pool (mean age = 30.12, SD = 11.15, female = 400, male = 110, diverse = 1). The derivation sample is part of an ongoing longitudinal survey into the mental health consequences of the pandemic. The subset of individuals who provided data for a second time (*n* = 318) formed our longitudinal validation sample (mean age = 30.54, SD = 11.28, female = 249, male = 68, diverse = 1). There were no significant differences between derivation sample and longitudinal validation sample in terms of age, sex, marital status, ethnicity, or employment status (*p* > .05). A second cross-validation sample consisted of 113 psychiatric inpatients (mean age = 43.93, SD = 14.64, female = 55, male = 58) recruited from the LMU Biobank study.

### Procedure

Non-clinical participants were recruited online via social media advertisements (Facebook) and university mailing lists to participate in the survey including the CoPaQ and other questionnaires (see below). The survey was run using the secure online LimeSurvey software. A forced response format was applied and only complete responses were included in the current analyses (*n* = 592). We excluded participants who gave incorrect responses to more than one out of three included bogus items (e.g., “Please, indicate completely agree”; *n* = 47) and with response times < 25 min, which we considered highly unlikely (median completion duration = 48 min; interquartile range [IQR] = 38–60; *n* = 7). At the end, by entering their email addresses participants had the opportunity to be included in a prize draw and take part in the 10-week follow-up assessment. Those participants at the 10-week follow-up time point who had response times < 15 min, which we considered highly unlikely (median completion duration = 29 min, IQR = 23–39.5; *n* = 7), were additionally excluded.

Psychiatric inpatients were recruited as part of the LMU Biobank study from the Department of Psychiatry and Psychotherapy of the LMU University Hospital Munich. Participants filled out the CoPaQ and other questionnaires (see below) using paper-pencil (*n* = 144). Exclusion criteria comprised an insufficient comprehension of German, an acute psychotic or manic episode, or acute suicidality. Furthermore, psychiatric inpatients’ responses were excluded if they had more than 10% missing data on each of the self-report questionnaires (*n* = 31). Missing values were then imputed using the *missForest* package [17] for non-parametric, iterative random-forest based imputation, which resulted in an imputation error of Out-of-bag_PFC_ = 0.1748.

To ensure data integrity, careless responders (longest or average length of consecutive identical responses was ±3 SD of the respective sample mean) in the derivation sample (*n* = 27), longitudinal- (*n* = 5), and psychiatric validation samples (*n* = 0) were excluded from further analyses using the *Careless* package [18]. The final sample size of the derivation sample was *n* = 511, of the longitudinal validation sample *n* = 355, and of the psychiatric inpatient sample *n* = 113.

### Other measures

#### Depression, Anxiety and Stress Scales-21 (DASS-21)

The total score of the German version of DASS-21 [19, 20] was included, which assesses psychological distress during the past week. Items are rated on a Likert scale of 0 (did not apply to me at all) to 3 (applied to me very much or most of the time). Higher scores indicate greater distress (range 0–63). In clinical and non-clinical samples good psychometric properties of the DASS-21 have been reported [21].

#### Revised-Green et al. Paranoid Thoughts Scale (R-GPTS)

Paranoid ideations over the past fortnight were assessed with the total score of the German version of the 18-item R-GPTS [22, 23]. Items are rated on a 5-point Likert scale ranging from 0 (not at all) to 4 (totally). Higher scores indicate higher levels of paranoia (score range 0–72). Excellent psychometric properties of the scales have been reported for the English version [22].

#### WHO (Five) Well-Being Index (WHO-5)

Well-being over the past 2 weeks was assessed by the German version of the WHO-5 [24, 25]. Items are rated on a 6-point Likert scale ranging from 0 (not present) to 5 (constantly present), with higher scores indicating greater well-being (score range: 0–30). Good psychometric properties have been reported in previous research [26].

### Statistical analyses

All analyses were conducted in *R* v4.0.3 [27].

Descriptive statistics and associations between variables were tested using bivariate Pearson’s correlation coefficients, Chi-square tests (χ^2^), and unpaired two-sample *t* tests (Welch t-test) when appropriate. We report magnitudes of effect sizes of 0.10 considered “small”, those of 0.30 as “medium”, and those of 0.50 as “large” according to Cohen [28].

We conducted exploratory factor analysis (EFA) based on polychoric correlations with the maximum likelihood estimator (ML) and oblimin rotation to assess the structure of items and refine the item pool by deleting poor-fitting items using the *Psych* package [29]. Items were then considered for deletion one at a time during EFA based on factor loadings (not loading higher than 0.30 on any factor, or loadings above 0.30 on more than one factor), communalities (<.30), content of items (e.g., theoretically inconsistent or redundant), item dependencies, sharp drop in item loading, and differences in response scale. In addition, items with an overall endorsement of < 10% across the derivation sample, longitudinal- and psychiatric validation samples were deleted. The number of factors to extract was determined through Empirical Kaiser Criterion (EKC), parallel analysis using polychoric correlations, and ML discrepancy function.

To validate the factor structure of the selected items per subscale, confirmatory factor analysis (CFA) with weighted least square mean and variance adjusted (WLSMV) estimator was conducted in the longitudinal and psychiatric validation samples using the *lavaan* package [30]. Model fit was assessed using the Comparative Fit Index (CFI; ≤.95 considered as acceptable) and Root Mean Square Error of Approximation (RMSEA; ≤ .08 considered as acceptable) following common recommendation [31]. Items, which loaded poorly on the factors in both validation samples, were deleted to arrive at the final version of the respective subscales of the questionnaire. Finally, we used modification indices to identify the best fitting model. Internal consistency of the different subscales with more than two items was determined by calculating McDonald’s Omega (ω) and Cronbach’s Alpha (α) using the *MBESS* package (v4.8.0; [32]).

Where appropriate, criterion and construct validity were established by testing differences (using Welch two sample t-tests) and strength of associations (using Pearson’s *r*) in the derivation sample, respectively. To evaluate criterion validity of COVID-19 mental health impact subscales, we assessed if these subscales were associated with self-reported lifetime mental health diagnosis. In terms of construct validity, the COVID-19 mental health impact-, positive coping-, conspiracy-, and institutional & political trust subscale scores were correlated with different mental health outcome scores that related to psychological distress (DASS-21; [19]), psychological well-being (WHO-5;) [24], and paranoia (R-GPTS;) [22]).

## Study Part 2: Research application

## Methods

### Matching

To obtain a more comparable case-control sample for our research application example, the clinical and non-clinical samples were matched on age, sex, and employment status using *R* software and the *MatchIt* (v4.1.0) package [33]. After matching, clinical and non-clinical samples were comparable in age and sex (age: t(221.65) = − 0.58, *p* = 0.564; sex: χ^2^(2) = 1.37, *p* = 0.505), but differences remained for employment status (χ^2^(6) = 21.98, *p* = 0.001).

### Measures

Following validation of the CoPaQ, we selected the subscales inquiring about perceived countermeasure necessity and countermeasure compliance as well as COVID-19 contamination anxiety, COVID-19 institutional & political trust, COVID-19 conspiracy beliefs, and COVID-19 physical health risk factors for our research application example. We further included data on the DASS-21 anxiety subscale and refer to this as ‘general anxiety’ throughout the manuscript to demarcate this construct from ‘COVID-19 contamination anxiety’. Finally, we assessed paranoia using the R-GPTS total score.

### Assessment of group differences

We conducted Welch two sample t tests and calculated standardised mean differences (SMD) to assess group differences in support of COVID-19 related governmental restrictions and recommendations regarding perceived countermeasure necessity and countermeasure compliance as well as COVID-19 contamination anxiety, COVID-19 institutional & political trust, COVID-19 conspiracy beliefs, COVID-19 physical health risk factors, general anxiety, and paranoia. To assess robustness of results, also against violations of homoscedasticity, we provide 95% bootstrapped confidence intervals (95% CI) of the SMD values using 5000 bootstrapped samples with replacement. All hypothesis testing was two-tailed according to α = 0.05.

### Correlation analysis

To explore the strength of statistical association between support of public health directives and COVID-19 contamination anxiety, COVID-19 institutional & political trust, COVID-19 conspiracy beliefs, COVID-19 physical health risk factors, general anxiety, and paranoia in clinical and non-clinical samples separately, we performed bivariate Spearman’s rho (*ρ*) correlation analyses and tested whether the strength of associations differed between the clinical and non-clinical group by conducting Fisher Z transformations with adapted standard errors for Spearman’s *ρ* [34].

## Study Part 1: Questionnaire validation

## Results

### Descriptive statistics

We provide participant characteristics of the derivation sample, longitudinal validation sample, and psychiatric validation sample in Table 1. The majority of the psychiatric inpatient sample had received a clinician’s confirmed clinical diagnosis based on the International Statistical Classification of Diseases and Related Health Problems, 10th revision (ICD-10) criteria of depression, substance abuse disorder, personality disorder, and anxiety disorder. Comorbidity was high with more than two-third of patients meeting criteria for more than one psychiatric diagnosis (see Supplementary Table 1 for more details).

**Table 1:** Socio-demographics and baseline characteristics of the derivation sample, longitudinal validation sample, and psychiatric inpatient sample

	Derivation sample (*n* = 511)	Longitudinal sample (*n* = 318)	Psychiatric sample (*n* = 113)
Age, *mean (SD)*	30.12 (11.15)	30.54 (11.28)	43.93 (14.64)
Men sex, *n*	110	68	58
Employment status, *n (%)*			
Full-time employed	114 (22.3)	73 (23.0)	32 (28.3)
Part-time employed	72 (14.1)	47 (14.8)	15 (13.3)
Self-employed	16 (3.1)	11 (3.5)	4 (3.5)
Student	248 (48.5)	157 (49.4)	11 (9.7)
Retired	8 (1.6)	4 (1.3)	17 (15.0)
Caregiver	2 (0.4)	0 (0.0)	0 (0.0)
Not employed	14 (2.7)	8 (2.5)	26 (23.0)
Other	37 (7.2)	18 (5.7)	8 (7.1)
Self-reported lifetime diagnoses, *n (%)*			
Diagnostic categories			
Depressive Disorders	120 (23.5)	77 (24.2)	93 (82.3)
Bipolar Disorders	5 (1.0)	3 (0.9)	11 (9.7)
Psychotic Disorders	3 (0.6)	0 (0.0)	17 (15.0)
Anxiety Disorders	62 (12.1)	36 (11.3)	30 (26.5)
Post-Traumatic Stress Disorder	26 (5.1)	17 (5.3)	18 (15.9)
Obsessive-Compulsive and Related Disorders	7 (1.4)	7 (2.2)	6 (5.3)
Eating Disorders	28 (5.5)	14 (4.4)	17 (15.0)
Substance-Related and Addictive Disorders	9 (1.8)	3 (0.9)	31 (27.4)
Attention-Deficit/Hyperactivity Disorder	15 (2.9)	8 (2.5)	6 (5.3)
Somatoform Disorders	4 (0.8)	2 (0.6)	7 (6.2)
Personality Disorders	14 (2.7)	7 (2.2)	22 (19.5)
Autism Spectrum Disorder	4 (0.8)	3 (0.9)	8 (7.1)
Dementia	0 (0.0)	0 (0.0)	2 (1.8)

n indicates the number of participants

*SD* Standard Deviation

### Exploratory and confirmatory factor analyses

Standardised oblimin rotated factor loadings of the final items are presented in Table 2. Model fit indices and internal consistency estimates of the respective subscales are presented in Tables 3 and 4, respectively. An overview of item selection decisions and related criteria for each subscale can be found in the Supplementary Material. Items loading on the respective subscales can be summed for further analyses.

**Table 2:** Final items and factor loadings from EFA and CFAs in our three samples

	Derivation sample	Longitudinal validation sample	Psychiatric validation sample
	EFA Loadings	CFA Loadings	CFA Loadings
**COVID-19 contamination anxiety**			
I will infect myself with COVID-19	0.54	0.54	0.54
Please indicate how likely you think it is that you will be infected with COVID-19.	0.74	0.58	0.77
people close to me are infected with COVID-19.	0.88	0.89	0.65
I will infect other people with COVID-19.	0.79	0.72	0.73
**COVID-19 hygiene measures**			
keeping at least 1.5 m distance from other people	0.67	0.67	0.63
coughing or sneezing into the crook of your arm or into a handkerchief	0.71	0.52	0.54
not touching mouth, eyes or nose with hands	0.81	0.80	0.58
regular washing of hands	0.93	0.79	0.63
washing hands extensively (for at least 30 s)	0.92	0.82	0.79
increased disinfection of hands and objects.	0.68	0.65	0.68
**COVID-19 social distancing**			
cancelling private meetings and family visits	0.82	0.79	0.76
cancelling trips to other cities	0.90	0.85	0.69
avoiding visits to canteens and restaurants	0.87	0.79	0.82
avoiding touching (e.g. shaking hands or hugging) when greeting or saying goodbye to other people	0.78	0.74	0.47
moving your work to home office	0.71	0.64	0.39
**COVID-19 anxiety buying**			
soap, detergent, cleaning products, washing powder, etc.	0.88	0.84	0.89
food (vegetables, lentils, rice, pasta...)	0.94	0.88	0.95
water (20 l per person)	0.79	0.73	0.85
toilet paper	0.84	0.82	0.92
cash	0.59	0.53	0.62
**COVID-19 political restrictions**			
temporary closures of kindergartens, schools and universities	0.84	0.80	0.85
temporary border closures	0.74	0.67	0.71
temporary closures of playgrounds	0.85	0.75	0.90
temporary closure of bars, pubs, theatres, cinemas, etc.	0.87	0.78	0.72
temporary curfews	0.80	0.76	0.82
**COVID-19 solidarity-based behaviours**			
donating blood	0.63	0.70	0.54
supporting people at risk, such as shopping for them or staying at home to protect people at risk to protect people at risk	0.86	0.77	0.88
supporting people who are experiencing existential hardship due to the current situation	0.87	0.79	0.86
offering help to close friends and family members	0.81	0.71	0.57
donating blood	0.77	0.80	0.65
**COVID-19 post-traumatic stress disorder symptoms**			
have had upsetting dreams that replay part of the experience of the COVID-19 pandemic or are clearly related to it	0.51	0.61	0.60
have had powerful images or memories that sometimes come into my mind in which I feel the experience of the COVID-19 pandemic is happening again in the here and now	0.75	0.72	0.75
have avoided internal reminders of the experience of the COVID-19 pandemic (e.g. thoughts, feeling, or physical sensations)	0.97	0.82	0.64
have avoided external reminders of the experience of the COVID-19 pandemic (e.g. people, places, conversations, objects, activities, or situations)	0.92	0.73	0.79
have been “super-alert”, watchful, or on guard	0.45	0.56	0.67
**COVID-19 sleep disturbances**			
difficulty falling asleep (< 30 min)	1.02	0.82	0.88
difficulty sleeping through the night	0.77	0.87	0.79
early morning awakening	0.70	0.74	0.74
**COVID-19 substance use**			
have consumed substantially more alcohol than usual.	0.68		
have smoked considerably more cigarettes than usual	0.86	0.61	0.66
have consumed considerably more drugs (e.g. tranquilizers, sleeping pills or stimulants) than usual	0.63	0.63	0.61
have felt a strong desire to consume addictive substances (alcohol, cigarettes, drugs)	0.87	0.90	0.84
have not been able to control my use of addictive substances (alcohol, cigarettes, drugs)	0.90	0.58	0.50
**COVID-19 daytime structure**			
have maintained a regular daily routine	0.90	0.81	0.76
have planned the day as detailed as possible	0.80	0.79	0.95
have integrated sports and exercise into my daily life	0.39	0.55	0.58
**COVID-19 social contacts**			
have maintained my social contacts (telephone, visits or video chats)	0.47	0.68	0.65
have enjoyed the time together with people close to me	0.74	0.50	0.50
**COVID-19 inner resources**			
have sought stability in faith and/or religion	0.45		
have focused on my inner strengths, resources, abilities and talents	0.59	0.87	0.72
have changed my attitudes about what is really important to me in life	0.64	0.39	0.70
**COVID-19 political and institutional trust**			
have had the feeling that the political leadership was standing up for me	0.84	0.81	0.67
have perceived democracy as an effective form of government	0.85	0.75	0.77
have had the feeling that public institutions (e.g. police, judiciary) can be relied upon	0.78	0.79	0.82
have had the feeling that news and reports on the COVID-19 pandemic are being deliberately withheld	0.84	0.86	0.30
have perceived politicians as trustworthy	0.65	0.61	0.77
**COVID-19 conspiracy beliefs**			
have had the feeling that false reports or untruths about the COVID-19 pandemic are being deliberately disseminated on public broadcasting (e.g. radio and television stations).	0.62	0.49	0.62
have had the belief that there are alternative or secret explanations for current events	0.80	0.89	0.84
have had the belief that there is a relation between what is happening and the production and testing of biological weapons	0.88	0.72	0.77
have had the belief that what is happening here is the effect of a struggle or competition between different superpowers	0.95	0.77	0.84
have had the belief that this infection serves to deliberately reduce the world population, since there are no longer enough resources for everyone	0.85	0.64	0.88
**COVID-19 social cohesion**			
there is greater solidarity and cohesion in our society.	0.84	0.79	
I am an integral part of our society or community.	0.61	0.61	
our nation is growing closer together.	0.93	0.79

**Table 3:** Model fit indices of the CFA analyses in the longitudinal validation sample

	Longitudinal validation sample	Psychiatric validation samples		
Subscale	CFI	RMSEA	CFI	RMSEA
COVID-19 contamination anxiety	0.996	0.039	1.000	< 0.001
COVID-19 hygiene measures	0.924	0.069	1.000	< 0.001
COVID-19 social distancing	1.000	< 0.001	0.979	0.061
COVID-19 anxiety buying	0.951	0.081	0.991	0.056
COVID-19 political restrictions	0.993	0.045	0.955	0.107
COVID-19 solidarity-based behaviours	0.988	0.037	1.000	< 0.001
COVID-19 mental health subscale	0.938	0.038	0.994	0.011
COVID-19 positive coping	0.979	0.049	0.965	0.066
COVID-19 institutional & political trust	0.931	0.138	0.997	0.027
COVID-19 conspiracy beliefs	0.924	0.069	0.880	0.119
COVID-19 social cohesion	1.000	< 0.001	n.a.	n.a.

*CFI* Comparative Fit Index, *RMSEA* Root Mean Square Error of Approximation, *n.a.* Heywood case

**Table 4:** Internal consistency estimates of the different CoPaQ subscales based on McDonald’s Omega and Cronbach’s Alpha in our three samples

	Derivation sample		Longitudinal validation sample		Psychiatric validation sample	
Subscale	*ω*	α	*ω*	α	*ω*	α
COVID-19 contamination anxiety	0.79	0.78	0.79	0.77	0.77	0.77
COVID-19 hygiene measures	0.83	0.83	0.86	0.84	0.81	0.81
COVID-19 social distancing	0.88	0.87	0.87	0.87	0.76	0.75
COVID-19 anxiety buying	0.86	0.85	0.88	0.87	0.93	0.92
COVID-19 political restrictions	0.88	0.88	0.86	0.86	0.90	0.90
COVID-19 solidarity-based behaviours	0.82	0.82	0.87	0.86	0.81	0.81
COVID-19 countermeasure compliance	n.a.	n.a.	n.a.	n.a.	n.a.	n.a.
COVID-19 PTSD symptoms	0.78	0.78	0.83	0.81	0.82	0.82
COVID-19 sleep disturbance	0.83	0.81	0.87	0.85	0.85	0.84
COVID-19 substance abuse	0.77	0.75	0.80	0.77	0.76	0.74
COVID-19-specific stressor impact	n.a.	n.a.	n.a.	n.a.	n.a.	n.a.
COVID-19 daytime structure	0.72	0.69	0.76	0.75	0.80	0.79
COVID-19 social contacts	n.a.	n.a.	n.a.	n.a.	n.a.	n.a.
COVID-19 inner strength	n.a.	n.a.	n.a.	n.a.	n.a.	n.a.
COVID-19 institutional & political trust	0.87	0.86	0.88	0.88	0.81	0.79
COVID-19 conspiracy beliefs	0.82	0.82	0.79	0.78	0.90	0.89
COVID-19 social cohesion	0.80	0.79	0.77	0.76	n.a.	n.a.

*ω* indicates McDonald’s Omega. α indicates Cronbach’s Alpha

*n.a.* not applicable

#### Study populations characteristics

From the first section of the CoPaQ, which aims at characterising participant under study, we decided to omit the answer option “Don’t know”. We further removed optional questions relating to psychotherapy via telephone or video platforms from the final questionnaire version.

#### COVID-19 contamination anxiety

Following criteria of poor fitting items (see Methods section), we deleted two items due to redundancy with similarly worded items. The EKC and parallel analysis indicated a 2-factor solution. During EFA, three additional items were deleted due to low factor loadings, factor cross-loadings and only one item loading onto the second factor. Therefore, a 1-factor solution was tested in a next step and all items were retained in the final model. The factor entailed items related to COVID-19 contamination anxiety (e.g., “I am worried I will infect myself with COVID-19”). Subsequently, CFAs in the longitudinal- and psychiatric validation samples were conducted using the 1-factor 4-item model identified during EFA, which showed a good model fit.

#### COVID-19 necessity of and compliance with countermeasures

Theoretical considerations, the EKC, and parallel analysis favoured a 1-factor solution for each of the respective COVID-19 countermeasures. During EFA all items were retained. CFAs in the longitudinal- and psychiatric validation samples provided good to acceptable model fit for the respective COVID-19 hygiene measures- (e.g., “regular washing of hands”), social distancing- (e.g., “cancelling private meetings and family visits”), anxiety buying- (e.g., “soap, detergent, cleaning products, washing powder, etc.”), political restrictions- (e.g., “temporary closure of bars, pubs, theatres, cinemas, etc.”), and solidarity-based behaviours (e.g., “offering help to close friends and family members”) subscales. The three items assessing COVID-19 countermeasure compliance of hygiene measures, social distancing, and curfews were grouped into an overall index since they were relatively independent from each other.

#### COVID-19 mental health impact

Poor fitting items were deleted due to poor content fit, dependency or redundancy. The EKC and parallel analysis indicated a three-factor solution. EFA suggested good model fit. Only one additional item was deleted due to high factor cross-loadings. Thereafter, all items were retained in the final model. The first factor entailed items related to COVID-19 post-traumatic stress disorder (PTSD) symptoms (e.g., “have had powerful images or memories that sometimes come into my mind in which I feel the experience of the COVID-19 pandemic is happening again in the here and now”), the second factor depicted COVID-19 sleep disturbance symptoms (e.g., “difficulty sleeping through the night”), and the third factor entailed items related to COVID-19 substance abuse (e.g., “have smoked considerably more cigarettes than usual”). Subsequently, CFAs in the longitudinal- and psychiatric validation samples were conducted using the 3-factor 13-item model identified during EFA, which did not provide a good model fit. Modification indices indicated dropping one additional poor fitting item, which was removed and the CFAs repeated. Now model fit indices suggested mixed results in the longitudinal validation sample with good to only adequate model fit according to RMSEA and CFI, respectively. Model fit in the psychiatric validation sample was good.

#### COVID-19-specific stressors impact

First, the not applicable answer option was recoded as zero and removed from in the final validated questionnaire version. Items with poorly fitting content were then deleted. In addition, items related to the ability to distance oneself from the stressors were deleted due to item dependency. As each stressor (e.g., “childcare”, “being in quarantine” or “being in home office”) can occur relatively independently, no factor analysis was applied for COVID-19-specific stressors, so the remaining items can be summed to an index.

#### COVID-19 positive coping

Three items were deleted due to poor item-scale content fit. The EKC and parallel analysis indicated a three-factor solution, which we evaluated in the subsequent EFA. During EFA, one additional item was deleted due to low factor loadings. The first factor entailed items related to keeping a daytime structure (e.g., “have planned the day as detailed as possible”), the second factor depicted positive coping items in terms of social contacts (e.g., “have maintained my social contacts (telephone, visits or video chats)”), and the third factor entailed items related to inner strength (e.g., “have changed my attitudes about what is really important to me in life”) during the pandemic. Subsequently, CFAs in the longitudinal- and psychiatric validation samples were conducted using the 3-factor 9-item model identified during EFA, which resulted in a poor model fit. Two additional items from the social contacts and inner strength subscales were deleted due to poor model and content fit. The subsequent model fit was good across samples.

#### COVID-19 institutional & political trust

First, we deleted one item due to item content. One item was reversed coded. The EKC and parallel analysis indicated a one-factor solution. During EFA all items were retained and related to political and institutional trust (e.g., “have had the feeling that the political leadership was standing up for me”). Subsequently, CFAs in the validation samples were conducted using the 1-factor 5-item model identified during EFA, which provided a poor model fit in the longitudinal validation sample and good model fit in the psychiatric validation sample.

#### COVID-19 conspiracy beliefs

We deleted one item due to a different response format. The EKC and parallel analysis indicated a 1-factor solution. During EFA all items were retained. The factor entailed items related to COVID-19 conspiracy beliefs (e.g., “have had the belief that what is happening here is the effect of a struggle or competition between different superpowers”). Subsequently, CFAs in our validation samples were conducted using the 1-factor 5-item model identified during EFA, which provided an acceptable model fit in the longitudinal but not in the psychiatric validation sample.

#### COVID-19 social cohesion

First, three items were removed due to item redundancy. The EKC and parallel analysis indicated a one-factor solution. During EFA all items were retained and related to COVID-19 social cohesion (e.g., “our nation is growing closer together”). Subsequently, CFAs in our validation samples were conducted using the 1-factor 3-item model identified during EFA, which provided a good model fit in the longitudinal sample, but resulted in model misspecification in the psychiatric validation sample.

#### Omitted subscales

The theoretically constructed subscales of COVID-19 media use (e.g., “have carried out an increased amount of research about the COVID-19 pandemic via the Internet”), COVID-19 interpersonal conflicts (e.g., “have had more physical arguments (e.g. beating, boxing, kicking) with people close to me”), and COVID-19 paranoid ideations (e.g., “have had the belief that the corona-virus was introduced to get at people like me”) were omitted from the CoPaQ measure due to poor model fit during EFA.

### Internal consistency

Overall, the CoPaQ subscale factors’ internal consistency estimates ranged from acceptable to excellent in our derivation-, longitudinal- and psychiatric samples.

### Construct and criterion validity

The COVID-19-specific stressor and mental health impact subscales were associated with all mental health outcomes and most strongly with greater psychological distress; the COVID-19 positive coping subscales were most strongly associated with greater psychological well-being; and the COVID-19 institutional & political trust and COVID-19 conspiracy beliefs subscales were most strongly associated with lower and higher paranoia levels, respectively (see Table 5). Table 6 shows the results for the evaluation of criterion validity, which shows that the COVID-19 mental health impact subscales were more strongly endorsed if the participant had self-reported a lifetime mental health diagnosis.

**Table 5:** Correlations between CoPaQ subscale scores and mental health outcomes

CoPaQ subscales	DASS-21 Total	WHO-5 Total	R-GPTS Total
COVID-19-specific stressor impact	.49**	−.41**	.34**
COVID-19 mental health impact			
PTSD symptoms	.42**	−.32**	.34**
Sleep disturbance	.47**	−.37**	.30**
Substance abuse	.29**	−.26**	.12**
COVID-19 positive coping			
Daytime structure	−.19**	.33**	−.05
Social contacts	−.33**	.40**	−16**
Inner strength	−.18**	.32**	.04
COVID-19 institutional & political trust	−.11*	.14**	−.13**
COVID-19 conspiracy beliefs	.14**	−.05	.30**

*DASS-21* Depression, Anxiety, Stress Scales-21, *WHO-5* WHO-5 Well-being Index (WHO), *R-GPTS* Revised-Green et al. Paranoid Thoughts Scale

* indicates *p* < .05. ** indicates *p* < .01

**Table 6:** T-tests comparing CoPaQ mental health and disease worries subscale scores for relevant variables to establish criterion validity

		COVID-19 PTSD symptoms		COVID-19 sleep disturbance		COVID-19 substance use	
	n	Mean (SD)	t	Mean (SD)	t	Mean (SD)	t
Mental Health Diagnosis							
No	340	2.85 (3.51)	−2.20*	2.66 (3.37)	−4.54***	0.82 (1.90)	−3.30**
Yes	171	3.71 (4.46)		4.16 (3.59)		1.71 (3.27)	

*P* values based on Welch two sample t test

*SD* Standard Deviation

*p < .05, **p < .01, ***p < .001

## Study Part 2: Research application

## Results

### Assessment of group differences

Psychiatric inpatients indicated greater support of COVID-19 public health directives compared to non-clinical individuals in terms of the perception of necessity of hygiene measures (t(199.93) = − 2.84; *p* < 0.01, 95% CI_bootstrappedSMD_ = − 0.60, − 0.12), political restrictions (t(208.92) = − 3.23; *p* < 0.01; 95% CI_bootstrappedSMD_ = − 0.66, − 0.18), and overall compliance with countermeasures (t(201.13) = − 2.07; *p* = 0.04; 95% CI_bootstrappedSMD_ = − 0.52, − 0.01). No difference between groups was evident for perception of necessity of social distancing (t(198.19) = − 0.87; *p* = 0.38; 95% CI_bootstrappedSMD_ = − 0.37, 0.15).

Further, COVID-19 contamination anxiety, COVID-19 institutional & political trust, and COVID-19 conspiracy beliefs did not differ between groups, whereas psychiatric inpatients indicated higher levels of general anxiety and COVID-19 physical health risk factors compared to non-clinical individuals with medium to high effect sizes (see Table 7).

**Table 7:** Descriptive statistics and differences of the independent variables

	Clinical sample		Non-clinical sample				
Subscales	Mean (SD)	range	Mean (SD)	range	p	SMD	95% CI_bootstrapped_
DASS-21							
General anxiety	12.02 (9.98)	0–40	6.58 (6.88)	0–28	< 0.001***	−0.605	− 0.83, − 0.38
R-GPTS							
Paranoia	10.04 (13.10)	0–61	9.96 (9.57)	0–47	0.958	−0.007	− 0.25, 0.28
CoPaQ							
COVID-19 conspiracy beliefs	3.49 (4.76)	0–19	3.50 (4.10)	0–16	0.976	0.004	−0.25, 0.28
COVID-19 institutional & political trust	11.43 (4.88)	0–20	10.88 (6.20)	0–20	0.461	−0.098	−0.36, 0.16
COVID-19 physical health risk factors	0.98 (1.17)	0–5	0.62 (1.19)	0–7	0.022*	−0.304	−0.59, − 0.04
COVID-19 contamination anxiety	5.93 (3.46)	0–16	6.61 (3.94)	0–16	0.169	0.183	−0.07, 0.44

*SD* Standard Deviation, *SMD* Standardised Mean Difference, *CI*_*bootstrapped*_ Confidence Interval_bootstrapped_ (5000 times)

### Correlational analysis

Bivariate Spearman’s *ρ* correlations of support of public health directives with general anxiety, COVID-19 physical health risk factors, COVID-19 contamination anxiety, paranoia, COVID-19 institutional & political trust, and COVID-19 conspiracy beliefs are displayed in Table 8.

**Table 8:** Spearman’s ρ correlations in the clinical and non-clinical samples

	Clinical sample				Non-clinical sample			
	Perception of necessity of				Perception of necessity of			
Subscales	Hygiene measures	Social distancing	Political restrictions	Compliance	Hygiene measures	Social distancing	Political restrictions	Compliance
General anxiety	.14	.12	.31**	.15	.09	.06	.07	−.07
Paranoia	−.11	−.03	.05	−.01	−.09	−.02	.07	−.15
Institutional & political trust	.16	.19*	.20*	.23*	.64**	.60**	.61**	.53**
Contamination anxiety	.34**	.36**	.29**	.19*	.36**	.48**	.36**	.38**
Physical health risk factors	.07	.07	.06	.10	.02	−.12	−.18	−.08
Conspiracy beliefs	−.07	−.10	−.06	−.14	−.30**	−.40**	−.37**	−.30**

* indicates *p* < .05. ** indicates *p* < .01

Necessity and compliance of COVID-19 public health countermeasure were positively associated with COVID-19 contamination anxiety and COVID-19 institutional & political trust in the clinical (Spearman’s *ρ* coefficients ranged from .19 to .36) and non-clinical group (Spearman’s *ρ* coefficients ranged from .36 to .64). Here, the strengths of associations were observed to be stronger in the non-clinical than the clinical sample. The difference in correlations was significant for COVID-19 institutional & political trust (Fisher’s Z_Hygiene measures_ = − 4.30, *p* < 0.01; Z_Social distancing_ = − 3.61, *p* < 0.01; Z_Political restrictions_ = − 3.65, *p* < 0.01; Z_Compliance_ = − 2.56, *p* = 0.01) but not for contamination anxiety (Fisher’s Z_Hygiene measures_ = − 0.16, *p* = 0.87; Z_Social distancing_ = − 1.05, *p* = 0.29; Z_Political restrictions_ = − 0.56, *p* = 0.57; Z_Compliance_ = − 1.50, *p* = 0.13). General anxiety was only associated significantly with the perception of necessity of political restrictions in the psychiatric inpatient sample (*ρ*_Clinical sample_ = .31; ρ_Non-clinical sample_ = .07). However, this difference in strength of associations was not statistically significant (Z_Political restrictions_ = − 1.80, *p* = 0.07). In the non-clinical sample only, COVID-19 conspiracy beliefs were negatively associated with COVID-19 countermeasure necessity and compliance (absolute *ρ* coefficients ranged from 0.30 to 0.40). Fisher’s Z tests indicated that these associations were significantly stronger in the non-clinical than clinical group for the perception of necessity of social distancing (Z = 2.33, *p* = 0.02) and political restrictions (Z = 2.36, *p* = 0.02) but not for hygiene measures (Z = 1.72, *p* = 0.08) or overall compliance (Z = 1.21, *p* = 0.23). In both samples, evidence for associations with paranoia and COVID-19 physical health risk factors was either absent or very small (absolute *ρ* coefficients ranged from 0.01 to 0.18).

## Discussion

Understanding the psychosocial impact of the COVID-19 pandemic in different study populations has become an international priority. In this study, we report first findings from the assessment of psychiatric inpatients and non-clinical subjects using the CoPaQ tool that was designed to measure key psychosocial aspects of the pandemic including contamination anxiety, countermeasure necessity and compliance, mental health impact, COVID-19-specific stressor impact, social media usage, interpersonal conflicts, paranoid ideations, institutional & political trust, conspiracy beliefs, and social cohesion. The questionnaire was developed for application in different study populations, has been published on the Open Science Framework and is currently available in 11 languages. Here, we conducted a psychometric evaluation of the scale in its German version using data from a longitudinal sample of non-clinical individuals and psychiatric inpatients. Factor analyses indicated that 12 out of 16 extracted subscales showed acceptable to good model fit indices, internal consistency estimates and, where appropriate, construct and criterion validity in at least one validation sample. Therefore, these subscales were retained in the final version of the CoPaQ. Overall, the final version of the CoPaQ represents a valid measure that can help to better understand key aspects affected by the pandemic as illustrated by our research application example.

Psychometric validation in the longitudinal non-clinical and psychiatric validation samples demonstrated key strengths and limitations of individual CoPaQ subscales. The theoretically constructed ‘COVID-19 social media usage’, ‘COVID-19 interpersonal conflicts’, and ‘COVID-19 paranoid ideations’ subscales were omitted from the final questionnaire version due to poor psychometric properties during EFA. In the longitudinal validation sample, the CoPaQ subscales of ‘COVID-19 hygiene measures’, ‘COVID-19 anxiety buying’, ‘COVID-19 mental health’, and ‘COVID-19 conspiracy beliefs’ only showed at least acceptable model fit for one of two indices and ‘COVID-19 institutional & political trust’ had poor model fit overall, which questions the utility of these subscales for repeated measurement designs. Similarly, in the psychiatric validation sample the subscale of ‘COVID-19 political restrictions’ showed acceptable model fit only according to CFI but not RMSEA and poor model fit was observed for the subscales of ‘COVID-19 conspiracy beliefs’ and ‘COVID-19 social cohesion’ limiting their valid application for this study population. However, internal consistency estimates of all subscales ranged from acceptable to excellent across samples. Moreover, where applicable we observed evidence for construct and criterion validity for the subscales of ‘COVID-19-specific stressor impact’, ‘COVID-19 mental health impact’, ‘COVID-19 positive coping’, ‘COVID-19 institutional & political trust’, and ‘COVID-19 conspiracy beliefs’. Future research is needed to evaluate the psychometric properties of the CoPaQ in different languages/cultures and study populations of interest during the current pandemic (e.g., frontline health workers, vulnerable individuals with a physical condition at risk of a severe course of COVID-19, or caretakers).

In order to present a first use case for which new tools are required for addressing a research question specific for the COVID-19 pandemic, we investigated whether psychiatric inpatients may have lower compliance with preventive countermeasures as previously discussed by some authors [5, 6]. Contrary to this view, our results indicate that the support of public health directives to contain the spread of the coronavirus was indeed greater in psychiatric inpatients primarily admitted for major depressive-, substance abuse-, personality-, and anxiety disorders, compared to age-, sex-, and employment status matched non-clinical individuals. Results may be regarded as preliminary evidence against the hypothesis that higher SARS-Cov-2 infection rates in psychiatric patients are due to lower adherence to countermeasures [5, 6]. Findings from correlational analyses indicated that particularly trust in institutions & politics as well as contamination anxiety were associated with increased levels of support of public health directives during the pandemic in clinical- and non-clinical individuals, which is in line with previous research [7–10]. However, general anxiety was additionally associated with increased support of public health directives only in the clinical sample. This could indicate general anxiety is a putative driver of the increased reported support of countermeasures in psychiatric patients, that we observed in our sample. It is important to note, however, that these results are restricted by non-significant correlational differences between samples. Additionally, the psychiatric inpatient setting may explain part of our findings since non-acceptance and non-compliance may likely lead to hospital discharge, social pressure from hospital staff and fellow patients, and a greater uncontrollable risk of infection in a relatively crowded hospital environment. In addition, paper-pencil questionnaire completion in the psychiatric patient sample may have contributed to greater socially desirable responses compared to online completion in the non-clinical group. Moreover, our findings and conclusions may not be extended to other diagnoses (e.g., a sample consisting of patients with psychotic disorders only) or treatment settings (e.g., outpatients).

### Limitations

There are obvious methodological limitations of our study: First, we have assessed construct validity and criterion validity for some but not all subscales of the CoPaQ. Future studies should test whether the other subscales assess what they are intended to measure. Second, since we applied the German version of the CoPaQ, generalisability of results to other languages/cultures is limited, which needs to be addressed in future research. Wider distribution of the CoPaQ and its translated versions in our open science approach would leverage such transcultural studies. Third, CFAs were based on relatively small sample sizes, which may have affected the robustness of results. As such, replications in larger study cohorts are needed. Fourth, the clinical and non-clinical samples are unlikely to fully represent the populations from which they were drawn. Finally, research needs to assess the CoPaQ’s predictive validity, test-retest reliability, and conduct evaluations in other study populations.

## Conclusion

Notwithstanding these caveats, the CoPaQ is a comprehensive, yet relatively brief self-assessment tool that covers a broad spectrum of pressing psychosocial topics during the current COVID-19 pandemic. The scale has the potential to facilitate the investigation of psychosocial reactions to the pandemic and could help assess the impact of potential future epidemics and pandemics if adapted accordingly. Our use case highlights its potential to untangle complex psychosocial aspects regarding levels of support of COVID-19 countermeasures in psychiatric inpatients and non-clinical individuals. Our findings stress the importance of transparent public health communication to foster trust in institutions and politics as well as inform the public about the potential contagiousness of the coronavirus to increase acceptance and adherence with the different public health directives in clinical and non-clinical groups during the current pandemic.

## Supplementary Information

**Additional file 1. Online Supplementary Material** includes an overview of clinician’s ascertained psychiatric diagnoses, item selection procedure, and final questionnaire version. Table S1 - Clinician’s ascertained psychiatric diagnoses in the psychiatric inpatient sample based on ICD-10.

## Data Availability

The datasets used and/or analysed during the current study are available from the corresponding author on reasonable request.

## Abbreviations

CoPaQ: COVID-19 Pandemic Mental Health Questionnaire
OSF: Open Science Framework
EFA: Explorative factor analysis
CFA: Confirmatory factor analysis
WLSMV: Weighted least square mean and variance adjusted
CFI: Comparative Fit Index
RMSEA: Root Mean Square Error of Approximation
ML: Maximum likelihood
Welch t-test: Unpaired two-sample *t* tests
IQR: Interquartile range
SD: Standard deviation
SMD: Standardised mean differences
χ2: Chi-square tests
ω: McDonald’s Omega
α: Cronbach’s Alpha
CI: Confidence intervals
*ρ*: Spearman’s rho
ICD-10: International Statistical Classification of Diseases and Related Health Problems, 10th revision
PTSD: Post-traumatic stress disorder
DASS-21: Depression, Anxiety and Stress Scales-21
R-GPTS: Revised-Green et al. Paranoid Thoughts Scale
WHO-5: WHO (Five) Well-Being Index

## References

[1] Allington D, Duffy B, Wessely S, Dhavan N, Rubin J. Health-protective behaviour, social media usage and conspiracy belief during the COVID-19 public health emergency. Psychol Med. 2020;9:1-7. 10.1017/S003329172000224X. 10.1017/S003329172000224X PMC7298098 32513320

[2] Freeman D, Waite F, Rosebrock L, Petit A, Causier C, East A, Jenner L, Teale AL, Carr L, Mulhall S, Bold E. Coronavirus conspiracy beliefs, mistrust, and compliance with government guidelines in England. Psychol Med. 2020;21:1-3. 10.1017/S0033291720001890. 10.1017/S0033291720001890 PMC7264452 32436485

[3] Gao J, Zheng P, Jia Y, Chen H, Mao Y, Chen S, Wang Y, Fu H, Dai J (2020). Mental health problems and social media exposure during COVID-19 outbreak. PLoS One.

[4] Rek SV, Freeman D, Reinhard M, Bühner M, Keeser D, Padberg F. The COVID-19 Pandemic Mental Health Questionnaire (CoPaQ): introducing a comprehensive measure of the psychosocial impact of the current coronavirus crisis. Open Sci Framew. 2020.

[5] Taquet, M, Luciano, S, Geddes, JR, Harrison, PJ. Bidirectional associations between COVID-19 and psychiatric disorder: retrospective cohort studies of 62 354 COVID-19 cases in the USA. The Lancet Psychiatry. 2021;8(2):130–40. 10.1101/2020.08.14.20175190. 10.1016/S2215-0366(20)30462-4 PMC7820108 33181098

[6] Wang, Q, Xu, R,Volkow, ND. Increased risk of COVID 19 infection and mortality in people with mental disorders: analysis from electronic health records in the United States. World Psychiatry. 2021;20(1):124–30. 10.1002/wps.20806. 10.1002/wps.20806 PMC7675495 33026219

[7] Rubin GJ, Finn Y, Potts HWW, Michie S (2015). Who is sceptical about emerging public health threats? Results from 39 national surveys in the United Kingdom. Public Health.

[8] Cori L, Bianchi F, Cadum E, Anthonj C. Risk perception and covid-19. Int J Environ Res Public Health. 2020;17(9):3114. 10.3390/ijerph17093114. 10.3390/ijerph17093114 PMC7246460 32365710

[9] Harper CA, Satchell LP, Fido D, Latzman RD. Functional fear predicts public health compliance in the COVID-19 pandemic. Int J Ment Health Addiction. 2020;27:1–4. 10.1007/s11469-020-00281-5. 10.1007/s11469-020-00281-5 PMC7185265 32346359

[10] Wahl I, Kastlunger B, Kirchler E (2010). Trust in authorities and power to enforce tax compliance: an empirical analysis of the “Slippery Slope Framework”. Law Policy.

[11] American Psychiatric Association. Diagnostic and statistical manual of mental disorders. United States; 2013. p. 21.

[12] Cloitre M, Shevlin M, Brewin CR, Bisson JI, Roberts NP, Maercker A, Karatzias T, Hyland P (2018). The international trauma questionnaire: development of a self-report measure of ICD-11 PTSD and complex PTSD. Acta Psychiatr Scand.

[13] Brooks SK, Webster RK, Smith LE, Woodland L, Wessely S, Greenberg N, Rubin GJ (2020). The psychological impact of quarantine and how to reduce it: rapid review of the evidence. Lancet..

[14] Chong M-Y, Wang W-C, Hsieh W-C, Lee C-Y, Chiu N-M, Yeh W-C, Huang TL, Wen JK, Chen CL (2004). Psychological impact of severe acute respiratory syndrome on health workers in a tertiary hospital. Br J Psychiatry.

[15] Qiu J, Shen B, Zhao M, Wang Z, Xie B, Xu Y (2020). A nationwide survey of psychological distress among Chinese people in the COVID-19 epidemic: implications and policy recommendations. Gen Psychiatry.

[16] Wang C, Pan R, Wan X, Tan Y, Xu L, Ho CS, Ho RC (2020). Immediate psychological responses and associated factors during the initial stage of the 2019 coronavirus disease (COVID-19) epidemic among the general population in China. Int J Environ Res Public Health.

[17] Stekhoven DJ, Bühlmann P (2012). MissForest—non-parametric missing value imputation for mixed-type data. Bioinformatics..

[18] Yentes RD, Wilhelm F (2018). careless: Procedures for computing indices of careless responding. R Packag version.

[19] Henry JD, Crawford JR (2005). The short-form version of the Depression Anxiety Stress Scales (DASS-21): construct validity and normative data in a large non-clinical sample. Br J Clin Psychol.

[20] Nilges P, Essau C (2015). Die Depressions-Angst-Stress-Skalen. Schmerz.

[21] Antony MM, Cox BJ, Enns MW, Bieling PJ, Swinson RP (1998). Psychometric properties of the 42-item and 21-item versions of the Depression Anxiety Stress Scales in clinical groups and a community sample. Psychol Assess.

[22] Freeman D, Loe BS, Kingdon D, Startup H, Molodynski A, Rosebrock L, Brown P, Sheaves B, Waite F, Bird JC. The revised Green et al., Paranoid Thoughts Scale (R-GPTS): psychometric properties, severity ranges, and clinical cut-offs. Psych Med. 2021;51(2):244–53. 10.1017/S0033291719003155. 10.1017/S0033291719003155 PMC7893506 31744588

[23] Rek SV, Freeman D, Reinhard M, Bühner M, Padberg F. Psychometric evaluation of the German Revised-Green et al. Paranoid Thought Scales (R-GPTS) across clinical and non-clinical samples. In preparation. 10.1186/s12888-025-07538-0 PMC12625113 41254569

[24] Organization WH (1998). Wellbeing measures in primary health care/the DEPCARE project: report on a WHO meeting, Stockholm, Sweden 12-13 February 1998. Wellbeing measures in primary health care/the DEPCARE project: report on a WHO meeting, Stockholm, Sweden 12–13 February 1998.

[25] Brähler E, Mühlan H, Albani C, Schmidt S (2007). Teststatistische prüfung und normierung der deutschen versionen des EUROHIS-QOL lebensqualität-Index und des WHO-5 wohlbefindens-index. Diagnostica..

[26] Topp CW, Østergaard SD, Søndergaard S, Bech P (2015). The WHO-5 Well-Being Index: a systematic review of the literature. Psychother Psychosom.

[27] Team RC (2013). R: a language and environment for statistical computing.

[28] Cohen J (1988). Statistical power analysis for the behavioural sciences.

[29] Revelle W. psych: Procedures for psychological, psychometric, and personality research. R package version 2.1.6. Evanston: Northwestern University; 2021.

[30] Rosseel Y (2012). Lavaan: an R package for structural equation modeling and more. Version 0.5–12 (BETA). J Stat Softw.

[31] Hu L, Bentler PM (1999). Cutoff criteria for fit indexes in covariance structure analysis: conventional criteria versus new alternatives. Struct Equ Model Multidiscip J.

[32] Kelley K, Lai K, Lai MK, Suggests M (2020). Package ‘MBESS’.

[33] Ho D, Imai K, Imai MK (2018). Package ‘MatchIt.’ Version.

[34] Sheskin DJ (2004). Handbook of parametric and nonparametric statistical procedures. crc Press.

