# Supplementary material for: The COVID-19 Pandemic Mental Health Questionnaire (CoPaQ): psychometric evaluation and compliance with countermeasures in psychiatric inpatients and non-clinical individuals
Source: BMC Psychiatry. 2021 Aug 31;21:426. doi: 10.1186/s12888-021-03425-6 (PMC8406012; doi:10.1186/s12888-021-03425-6)
Supplement: Supplementary file 1 — Additional file 1. Online Supplementary Material includes an overview of clinician’s ascertained psychiatric diagnoses, item selection procedure, and final questionnaire version. Table S1 - Clinician’s ascertained psychiatric diagnoses in the psychiatric inpatient sample based on ICD-10. [file 12888_2021_3425_MOESM1_ESM.docx]

ONLINE SUPPLEMENTARY MATERIAL

**The** **COVID-19 Pandemic Mental Health Questionnaire (CoPaQ): Psychometric Evaluation and Compliance with Countermeasures in Psychiatric Inpatients and Non-clinical Individuals**

Stephanie V. Rek^1,2^, Markus Bühner^3^, Matthias A. Reinhard^1^, Daniel Freeman^4^, Daniel Keeser^1^, Kristina Adorjan^1^, Peter Falkai^1^, & Frank Padberg^1^

^1^Department of Psychiatry and Psychotherapy, LMU University Hospital Munich, Munich, Germany

^2^International Max Planck Research School for Translational Psychiatry (IMPRS-TP), Munich, Germany

^3^Department of Psychology, LMU Munich, Munich, Germany

^4^Department of Psychiatry, University of Oxford, Oxford, UK

Table of Contents

[SUPPLEMENTARY RESULTS 2](#_Toc68854873)

[Supplementary Table 1. Clinician’s ascertained psychiatric diagnoses in the psychiatric inpatient sample based on ICD-10 2](#_Toc68854874)

[CoPaQ Subscales Validation 3](#_Toc68854875)

[COVID-19 contamination anxiety 3](#_Toc68854876)

[COVID-19 necessity of and compliance with countermeasures 4](#_Toc68854877)

[COVID-19 mental health impact 5](#_Toc68854878)

[COVID-19-specific stressors impact 6](#_Toc68854879)

[COVID-19 positive coping 6](#_Toc68854880)

[COVID-19 interpersonal conflicts* 7](#_Toc68854881)

[COVID-19 media use* 7](#_Toc68854882)

[COVID-19 institutional & political trust 7](#_Toc68854883)

[COVID-19 paranoid ideations* 8](#_Toc68854884)

[COVID-19 conspiracy beliefs 8](#_Toc68854885)

[COVID-19 social cohesion 8](#_Toc68854886)

[Covid-19 Pandemic Mental Health Questionnaire (CoPaQ) - final version 9](#_Toc68854887)

# SUPPLEMENTARY RESULTS

## Supplementary Table 1. Clinician’s ascertained psychiatric diagnoses in the psychiatric inpatient sample based on ICD-10

| Clinician’s ascertained diagnoses | n (%) |
| --- | --- |
| Number of diagnoses |  |
| 0 | 0 (0) |
| 1 | 30 (26.54) |
| 2 | 43 (38.06) |
| 3 | 24 (21.24) |
| >= 4 | 16 (14.16) |
| Any diagnosis | 113 (100) |
| Diagnostic categories |  |
| Depressive Disorders (F32 - F34) | 82 (72.57) |
| Bipolar Disorders (F30, F31) | 12 (10.62) |
| Psychotic Disorders (F20, F22, F23, F25, F29) | 11 (9.73) |
| Anxiety Disorders (F40, F41) | 23 (20.35) |
| Post-Traumatic Stress Disorder (F43) | 19 (16.81) |
| Dissociative Disorders (F44) | 5 (4.42) |
| Obsessive-Compulsive and Related Disorders (F45) | 4 (3.53) |
| Eating Disorders (F50) | 5 (4.42) |
| Substance-Related and Addictive Disorders (F10 - F19) | 53 (46.90) |
| Attention-Deficit/Hyperactivity Disorder | 1 (0.88) |
| Somatoform Disorders (F45) | 6 (5.31) |
| Personality Disorders (F60, F61, F69) | 24 (21.23) |
| Autism Spectrum Disorder (F84) | 8 (7.07) |
| Organic Disorder (F06) | 3 (2.65) |
| Other (F70, F71, F79, F80, F84) | 5 (4.42) |

*Note*. n indicates the number of participants.

## CoPaQ Subscales Validation

### COVID-19 contamination anxiety

| *^b^I have no means of control over the COVID-19 pandemic. | |
| --- | --- |
| I will infect myself with COVID-19. | |
| Please indicate how likely you think it is that you will be infected with COVID-19. | |
| people close to me are infected with COVID-19. | |
| I will infect other people with COVID-19. | |
| *^a^the consequences of the COVID-19 pandemic will greatly affect me personally. | |
| *^a^in case of infection with COVID-19 the consequences for my health will be severe. | |
| *^h^I will die of COVID-19. | |
| *^g^people close to me will die of COVID-19. | |
| *Create sum score* |  |

Note. * = deleted item/subscale, a = item redundancy, b = low factor loading, c = item content, d = item dependency, e = answer scale, f = response frequency < 10%, g = item cross-loadings, h = only item loading on a factor, i = Heywood case, j = drop in item loading (r) = reversed scored.

### COVID-19 necessity of and compliance with countermeasures

| **COVID-19 hygiene measures** | |
| --- | --- |
| 1. keeping at least 1.5 metres distance from other people | |
| 1. coughing or sneezing into the crook of your arm or into a handkerchief | |
| 1. not touching mouth, eyes or nose with hands | |
| 1. regular washing of hands | |
| 1. washing hands extensively (for at least 30 seconds) | |
| 1. increased disinfection of hands and objects. | |
| *Create sum score* |  |
| **COVID-19 social distancing** | |
| 1. cancelling private meetings and family visits | |
| 1. cancelling trips to other cities | |
| 1. avoiding visits to canteens and restaurants | |
| 1. avoiding touching (e.g. shaking hands or hugging) when greeting or saying goodbye to other people | |
| 1. moving your work to home office | |
| *Create sum score* |  |
| **COVID-19 anxiety buying** | |
| 1. soap, detergent, cleaning products, washing powder, etc. | |
| 1. food (vegetables, lentils, rice, pasta...) | |
| 1. water (20 litres per person) | |
| 1. toilet paper | |
| 1. cash | |
| *Create sum score* |  |
| **COVID-19 political restrictions** | |
| 1. temporary closures of kindergartens, schools and universities | |
| 1. temporary border closures | |
| 1. temporary closures of playgrounds | |
| 1. temporary closure of bars, pubs, theatres, cinemas, etc. | |
| 1. temporary curfews | |
| *Create sum score* |  |
| **COVID-19 solidarity-based behaviours** | |
| 1. donating blood | |
| 1. supporting people at risk, such as shopping for them or staying at home to protect people at risk to protect people at risk | |
| 1. supporting people who are experiencing existential hardship due to the current situation | |
| 1. offering help to close friends and family members | |
| 1. getting involved in neighbourhood assistance | |
| *Create sum score* |  |
| To what extent have you adhered to the following COVID-19 pandemic measures over the past two weeks? | |
| 1. Hygiene measures | |
| 1. Reduction of social contacts | |
| 1. Curfews | |
| *Create sum score* |  |

Note. * = deleted item/subscale, a = item redundancy, b = low factor loading, c = item content, d = item dependency, e = answer scale, f = response frequency < 10%, g = item cross-loadings, h = only item loading on a factor, i = Heywood case, j = drop in item loading (r) = reversed scored.

### COVID-19 mental health impact

| **COVID-19 post-traumatic stress disorder symptoms** | |
| --- | --- |
| 1. have had upsetting dreams that replay part of the experience of the COVID-19 pandemic or are clearly related to it. | |
| 2. have had powerful images or memories that sometimes come into my mind in which I feel the experience of the COVID-19 pandemic is happening again in the here and now. | |
| 3. have avoided internal reminders of the experience of the COVID-19 pandemic (e.g. thoughts, feeling, or physical sensations). | |
| 4. have avoided external reminders of the experience of the COVID-19 pandemic (e.g. people, places, conversations, objects, activities, or situations). | |
| 5. have been “super-alert”, watchful, or on guard. | |
| *^g^6. have been feeling jumpy or easily startled. | |
| *Create sum score* |  |
| *^c^7. have suffered from unforeseeable severe anxiety attacks (panic) with physical symptoms (e.g. palpitations, chest pain, dizziness). | |
| **^d^Optional (if >0):* During such an anxiety attack I was afraid of dying, losing control or one’s mind. | |
| **COVID-19 sleep disturbance** | |
| 8. have suffered from sleep problems, such as | |
| 1. difficulty falling asleep (< 30 minutes) | |
| 1. difficulty sleeping through the night | |
| 1. early morning awakening | |
| *Create sum score* |  |
| *^a^9. fearful dreams or nightmares not about the COVID-19 pandemic | |
| *^a^10. fearful dreams or nightmares about the COVID-19 pandemic | |
| *^c^11. felt or behaved in a more irritable, rageful, angry | |
| **COVID-19 substance abuse** | |
| *^g^12. have consumed substantially more alcohol than usual. | |
| 13. have smoked considerably more cigarettes than usual. | |
| 14. have consumed considerably more drugs (e.g. tranquilizers, sleeping pills or stimulants) than usual. | |
| 15. have felt a strong desire to consume addictive substances (alcohol, cigarettes, drugs). | |
| 16. have not been able to control my use of addictive substances (alcohol, cigarettes, drugs). | |
| *Create sum score* |  |
| *^c^17. have had the excessive urge to wash and/or disinfect my hands again and again so that I do not become ill from germs or contamination. | |
| *^d^*optional:* I perceived the extent as nonsensical, distressing or excessive. | |
| *^c^18. have had the excessive urge to wash and/or disinfect my hands again and again so that I do not pass on germs or contamination to other people. | |
| *^d^*optional:* I perceived the extent as nonsensical, distressing or excessive. | |
| *^c^19. have visited my GP more often. | |
| *^c^20. have avoided visits to my GP. | |

Note. * = deleted item/subscale, a = item redundancy, b = low factor loading, c = item content, d = item dependency, e = answer scale, f = response frequency < 10%, g = item cross-loadings, h = only item loading on a factor, i = Heywood case, j = drop in item loading (r) = reversed scored.

### COVID-19-specific stressors impact

| 1. the current pandemic. |
| --- |
| 2. living in a small accommodation. |
| 3. being in quarantine. |
| 4. childcare. |
| 5. taking over school lessons. |
| 6. the curfew. |
| 7. being in home office. |
| 8. customer service. |
| 9. worries about my health. |
| 10. worries of not being able to get medical care. |
| *^c^11. worries about being sick with COVID-19 when I noticed first signs of symptoms such as fever, dry cough, breathing problems, sore throat, loss of smell/taste, headache or diarrhea. |
| 12. increased conflicts with people close to me. |
| 13. financial worries. |
| 14. uncertainties regarding my job, training place, studies or school. |
| *^c^15. concerns for my own personal safety. |
| *^c^16. concerns for the integrity of family members or friends. |
| 17. fears of what the future will bring, or that I won't be able to cope with everything. |
| *^c^18. thoughts that it would be better to be dead. |
| *Create sum score* |

Note. * = deleted item/subscale, a = item redundancy, b = low factor loading, c = item content, d = item dependency, e = answer scale, f = response frequency < 10%, g = item cross-loadings, h = only item loading on a factor, i = Heywood case, j = drop in item loading (r) = reversed scored.

### COVID-19 positive coping

| **Keeping a daytime structure** | |
| --- | --- |
| have maintained a regular daily routine. | |
| have planned the day as detailed as possible. | |
| have integrated sports and exercise into my daily life. | |
| *Create sum score* |  |
| **Social contacts** | |
| *^c^have had the opportunity to retreat to a private place. | |
| *^c^have reduced any contact with fellow human beings. | |
| have maintained my social contacts (telephone, visits or video chats). | |
| have enjoyed the time together with people close to me. | |
| *Create sum score* |  |
| *^c^have felt more hopeful that the corona-crisis will soon be over. | |
| **Inner strength** | |
| *^b^have sought stability in faith and/or religion. | |
| have focused on my inner strengths, resources, abilities and talents. | |
| have changed my attitudes about what is really important to me in life. | |
| *^b^have acknowledged and accepted the COVID-19 pandemic as reality. | |
| *Create sum score* |  |

Note. * = deleted item/subscale, a = item redundancy, b = low factor loading, c = item content, d = item dependency, e = answer scale, f = response frequency < 10%, g = item cross-loadings, h = only item loading on a factor, i = Heywood case, j = drop in item loading (r) = reversed scored.

### COVID-19 interpersonal conflicts*

| *^b^have had more conflicts with people close to me. |
| --- |
| *^a^have had more verbal arguments with people close to me. |
| *^c^have experienced becoming a victim of verbal abuse (e.g. threats, humiliations) with people close to me. |
| *^f^have had more physical arguments (e.g. beating, boxing, kicking) with people close to me. |
| *^f^have experienced becoming a victim of physical abuse (e.g. beating, boxing, kicking) by people close to me. |

Note. * = deleted item/subscale, a = item redundancy, b = low factor loading, c = item content, d = item dependency, e = answer scale, f = response frequency < 10%, g = item cross-loadings, h = only item loading on a factor, i = Heywood case, j = drop in item loading (r) = reversed scored.

### COVID-19 media use*

| *^c^have carried out an increased amount of research about the COVID-19 pandemic via the Internet. |
| --- |
| *^e^Please, indicate the approximate duration of involvement (media, Internet) with the coronavirus-topic in hours. |
| *^b^have tried to deliberately control my media consumption around COVID-19. |
| *^d^have succeeded in deliberately controlling my media consumption around COVID-19 (social media, television, internet). |
| *^b^have tried to avoid COVID-19 news and notifications as far as possible. |
| *^b^have felt burdened by medial images of or news reports about COVID-19. |
| *^a^have been able to distance myself mentally from news and notifications about COVID-19. (r) |

Note. * = deleted item/subscale, a = item redundancy, b = low factor loading, c = item content, d = item dependency, e = answer scale, f = response frequency < 10%, g = item cross-loadings, h = only item loading on a factor, i = Heywood case, j = drop in item loading (r) = reversed scored.

### COVID-19 institutional & political trust

| have had the feeling that the political leadership was standing up for me. | |
| --- | --- |
| have perceived democracy as an effective form of government. | |
| have had the feeling that public institutions (e.g. police, judiciary) can be relied upon. | |
| *^c^have worried about our economic development. | |
| have had the feeling that news and reports on the COVID-19 pandemic are being deliberately withheld. (r) | |
| have perceived politicians as trustworthy. | |
| *Create sum score* |  |

Note. * = deleted item/subscale, a = item redundancy, b = low factor loading, c = item content, d = item dependency, e = answer scale, f = response frequency < 10%, g = item cross-loadings, h = only item loading on a factor, i = Heywood case, j = drop in item loading (r) = reversed scored.

### COVID-19 paranoid ideations*

| *^h^have had the feeling that the rules we now need to follow are there to make my life miserable. | |
| --- | --- |
| *^h^have had the feeling that people looked at me as if I have got corona-virus. | |
| *^b^have had the feeling that people kept a greater distance from me specifically and more than was necessary. | |
| *^f^have had the belief that the virus is targeting me more than anyone else irrespective of my health. | |
| *^f^have had the belief that the corona-virus was introduced to get at people like me. | |
| *Create sum score* |  |

Note. * = deleted item/subscale, a = item redundancy, b = low factor loading, c = item content, d = item dependency, e = answer scale, f = response frequency < 10%, g = item cross-loadings, h = only item loading on a factor, i = Heywood case, j = drop in item loading (r) = reversed scored.

### COVID-19 conspiracy beliefs

| have had the feeling that false reports or untruths about the COVID-19 pandemic are being deliberately disseminated on public broadcasting (e.g. radio and television stations). | |
| --- | --- |
| have had the belief that there are alternative or secret explanations for current events. | |
| have had the belief that there is a relation between what is happening and the production and testing of biological weapons. | |
| have had the belief that what is happening here is the effect of a struggle or competition between different superpowers. | |
| have had the belief that this infection serves to deliberately reduce the world population, since there are no longer enough resources for everyone. | |
| *^c^thought that the crisis is not real, I am caught in a bad dream and just need to wake up. | |
| *^e^I’m convinced there’s a conspiracy behind many things in the world. | |
| *Create sum score* |  |

Note. * = deleted item/subscale, a = item redundancy, b = low factor loading, c = item content, d = item dependency, e = answer scale, f = response frequency < 10%, g = item cross-loadings, h = only item loading on a factor, i = Heywood case, j = drop in item loading (r) = reversed scored.

### COVID-19 social cohesion

| there is greater solidarity and cohesion in our society. | |
| --- | --- |
| I am an integral part of our society or community. | |
| our nation is growing closer together. | |
| *^a^there is less solidarity and cohesion in our society (e.g. excessive shopping, corona parties). (r) | |
| *^a^ I am not an integral part of our society or community. (r) | |
| *^a^ the crisis divides our public nation. (r) | |
| Create sum score |  |

Note. * = deleted item/subscale, a = item redundancy, b = low factor loading, c = item content, d = item dependency, e = answer scale, f = response frequency < 10%, g = item cross-loadings, h = only item loading on a factor, i = Heywood case, j = drop in item loading (r) = reversed scored.

# *Covid-19 Pandemic Mental Health Questionnaire (CoPaQ) - final version*

With the following questions we would like to learn about the personal and social **consequences of the COVID-19-pandemic (SARS-CoV-2)**.

First, we would like to ask you to answer the following initial questions.

| 1. Do you currently suffer from COVID-19 symptoms such as fever, dry cough, breathing problems, sore throat, loss of smell/taste, headaches or diarrhoea? | Yes | No |
| --- | --- | --- |
| 2. Have you been tested positive for SARS-CoV-2? | Yes | No |
| 3. Have you been tested positive for Anti-SARS-CoV-2 antibodies? | Yes | No |
| 4. Has someone close to you been infected with COVID-19? | Yes | No |
| 5. Has a person close to you died in the course of a COVID-19 disease? | Yes | No |
| 6. Has any of the people with whom you had direct contact in the past two weeks become infected with COVID-19? | Yes | No |
| 7. Please indicate which of the following risk factors for a severe course of COVID-19 apply to you. | | |
| 1. Older than 60 years | Yes | No |
| 1. Cardiovascular disease | Yes | No |
| 1. Diabetes | Yes | No |
| 1. Immunodeficiency, or taking medication that suppresses the immune system (e.g. cortisone) | Yes | No |
| 1. Chronic disease of the respiratory system (e.g. asthma, chronic bronchitis) | Yes | No |
| 1. Chronic liver disease | Yes | No |
| 1. Chronic kidney disease | Yes | No |
| 1. Acute cancer | Yes | No |
| 1. Cancer during past 5 years | Yes | No |
| 1. Long-standing heavy cigarette consumption (more than 20 cigarettes per day in the last 5-10 years) | Yes | No |
| 1. none of the above risk factors | Yes | No |
| 8. Please indicate which of the following risk factors for a severe course of COVID-19 apply to people living with you in a household. | | |
| 1. Older than 60 years | Yes | No |
| 1. Cardiovascular disease | Yes | No |
| 1. Diabetes | Yes | No |
| 1. Immunodeficiency, or taking medication that suppresses the immune system (e.g. cortisone) | Yes | No |
| 1. Chronic disease of the respiratory system (e.g. asthma, chronic bronchitis) | Yes | No |
| 1. Chronic liver disease | Yes | No |
| 1. Chronic kidney disease | Yes | No |
| 1. Acute cancer | Yes | No |
| 1. Cancer during past 5 years | Yes | No |
| 1. Long-standing heavy cigarette consumption (more than 20 cigarettes per day in the last 5-10 years) | Yes | No |
| 1. none of the above risk factors | Yes | No |

| 9. Please indicate whether you are currently in quarantine. | Yes | No |
| --- | --- | --- |
| 10. *if “Yes” to 9.:* Please indicate the number of days you have been in quarantine. |  | |
| 11. Please indicate whether you are currently under a state-imposed curfew. | Yes | No |
| 12. *if “Yes” to 11.:* Please indicate how many days you have been under the state-imposed curfew. |  | |
| 13. Please indicate if you are currently working remotely from home. | Yes | No |
| 14. *if “Yes” to 13.:* Please indicate how many days you have been working remotely from home. |  | |
| 15. Please indicate whether you are engaged in an essential activity for the maintenance of critical infrastructure in accordance with the emergency plan. | | |
| 1. doctors | Yes | No |
| 1. nurses | Yes | No |
| 1. clinical psychologists | Yes | No |
| 1. public safety and national security guards | Yes | No |
| 1. staff of local and national government | Yes | No |
| 1. supermarket vendors | Yes | No |
| 1. bakers | Yes | No |
| 1. professional cleaners | Yes | No |
| 1. if other, please indicate |  | |
| 16. Please indicate your employment status |  |  |
| 1. full-time employed | Yes | No |
| 1. part-time employed | Yes | No |
| 1. not employed | Yes | No |
| 1. self-employed | Yes | No |
| 1. caregiver (e.g., children, elderly) | Yes | No |
| 1. student | Yes | No |
| 1. retired | Yes | No |
| 1. other | Yes | No |
| 17. Please indicate your public health insurance coverage. | | |
| 1. None | | |
| 1. Partial coverage | | |
| 1. Full coverage (without psychiatric/psychotherapeutic care) | | |
| 1. Full coverage (with psychiatric/psychotherapeutic care) | | |
| 18. Please indicate whether you receive welfare benefits. | Yes | No |
| 19. Have you ever been diagnosed by a doctor or therapist with one or more of the following? | | |
| 1. Depression | Yes | No |
| 1. Mania/Bipolar disorder | Yes | No |
| 1. Psychotic disorders (including schizophrenia) | Yes | No |
| 1. Anxiety disorder | Yes | No |
| 1. Posttraumatic stress disorder | Yes | No |
| 1. Eating disorder | Yes | No |
| 1. Compulsive disorders (OCD) | Yes | No |
| 1. Substance abuse or Addiction disorder | Yes | No |
| 1. Attention disorder (ADD or ADHD) | Yes | No |
| 1. Somatoform disorder | Yes | No |
| 1. Personality disorder | Yes | No |
| 1. Autism Spectrum Disorder (including Asperger’s Syndrome) | Yes | No |
| 1. Cognitive disorder/dementia | Yes | No |
| 20. Are you currently receiving psychotherapy? | Yes | No |

How necessary and useful do you consider the following behaviour since the COVID 19 pandemic?

| Hygiene measures, such as | Not at all |  |  |  | Very much |
| --- | --- | --- | --- | --- | --- |
| 1. keeping at least 1.5 metres distance from other people | 0 | 1 | 2 | 3 | 4 |
| 1. coughing or sneezing into the crook of your arm or into a handkerchief | 0 | 1 | 2 | 3 | 4 |
| 1. not touching mouth, eyes or nose with hands | 0 | 1 | 2 | 3 | 4 |
| 1. regular washing of hands | 0 | 1 | 2 | 3 | 4 |
| 1. washing hands extensively (for at least 30 seconds) | 0 | 1 | 2 | 3 | 4 |
| 1. increased disinfection of hands and objects. | 0 | 1 | 2 | 3 | 4 |
| Reduction of social contacts, e.g. | | | | | |
| 1. cancelling private meetings and family visits | 0 | 1 | 2 | 3 | 4 |
| 1. cancelling trips to other cities | 0 | 1 | 2 | 3 | 4 |
| 1. avoiding visits to canteens and restaurants | 0 | 1 | 2 | 3 | 4 |
| 1. avoiding touching (e.g. shaking hands or hugging) when greeting or saying goodbye to other people | 0 | 1 | 2 | 3 | 4 |
| 1. moving your work to home office | 0 | 1 | 2 | 3 | 4 |
| Build up stocks, such as | | | | | |
| 1. soap, detergent, cleaning products, washing powder, etc. | 0 | 1 | 2 | 3 | 4 |
| 1. food (vegetables, lentils, rice, pasta...) | 0 | 1 | 2 | 3 | 4 |
| 1. water (20 litres per person) | 0 | 1 | 2 | 3 | 4 |
| 1. toilet paper | 0 | 1 | 2 | 3 | 4 |
| 1. cash | 0 | 1 | 2 | 3 | 4 |
| Political measures, such as | | | | | |
| 1. temporary closures of kindergartens, schools and universities | 0 | 1 | 2 | 3 | 4 |
| 1. temporary border closures | 0 | 1 | 2 | 3 | 4 |
| 1. temporary closures of playgrounds | 0 | 1 | 2 | 3 | 4 |
| 1. temporary closure of bars, pubs, theatres, cinemas, etc. | 0 | 1 | 2 | 3 | 4 |
| 1. temporary curfews | 0 | 1 | 2 | 3 | 4 |
| Solidarity-based behaviour, such as | | | | | |
| 1. donating blood | 0 | 1 | 2 | 3 | 4 |
| 1. supporting people at risk, such as shopping for them or staying at home to protect people at risk to protect people at risk | 0 | 1 | 2 | 3 | 4 |
| 1. supporting people who are experiencing existential hardship due to the current situation | 0 | 1 | 2 | 3 | 4 |
| 1. offering help to close friends and family members | 0 | 1 | 2 | 3 | 4 |
| 1. getting involved in neighbourhood assistance | 0 | 1 | 2 | 3 | 4 |

| To what extent have you adhered to the following COVID-19 pandemic measures over the past two weeks? | | | | | |
| --- | --- | --- | --- | --- | --- |
|  | Not at all |  |  |  | Very much |
| 1. Hygiene measures | 0 | 1 | 2 | 3 | 4 |
| 1. Reduction of social contacts | 0 | 1 | 2 | 3 | 4 |
| 1. Curfews | 0 | 1 | 2 | 3 | 4 |

**Risk perception**

How do you currently perceive the risk of the COVID-19 pandemic?

“I am worried that…”

|  | Not at all |  |  |  | Very much |
| --- | --- | --- | --- | --- | --- |
| I will infect myself with COVID-19. | 0 | 1 | 2 | 3 | 4 |
| Please indicate how likely you think it is that you will be infected with COVID-19 | 0 | 1 | 2 | 3 | 4 |
| people close to me are infected with COVID-19. | 0 | 1 | 2 | 3 | 4 |
| I will infect other people with COVID-19. | 0 | 1 | 2 | 3 | 4 |

The following is a list of statements that deal with the handling and impact of the COVID-19 Pandemic. Please indicate the extent to which the following statements have applied to you in the **past two weeks.**

"Because of the COVID-19 pandemic, **over the past 14 days** I..."

|  | Not at all |  |  |  | Very much |
| --- | --- | --- | --- | --- | --- |
| have had upsetting dreams that replay part of the experience of the COVID-19 pandemic or are clearly related to it. | 0 | 1 | 2 | 3 | 4 |
| have had powerful images or memories that sometimes come into my mind in which I feel the experience of the COVID-19 pandemic is happening again in the here and now. | 0 | 1 | 2 | 3 | 4 |
| have avoided internal reminders of the experience of the COVID-19 pandemic (e.g. thoughts, feeling, or physical sensations). | 0 | 1 | 2 | 3 | 4 |
| have avoided external reminders of the experience of the COVID-19 pandemic (e.g. people, places, conversations, objects, activities, or situations). | 0 | 1 | 2 | 3 | 4 |
| have been “super-alert”, watchful, or on guard. | 0 | 1 | 2 | 3 | 4 |
| have suffered from sleep problems, such as | 0 | 1 | 2 | 3 | 4 |
| 1. difficulty falling asleep (< 30 minutes) | 0 | 1 | 2 | 3 | 4 |
| 1. difficulty sleeping through the night | 0 | 1 | 2 | 3 | 4 |
| 1. early morning awakening | 0 | 1 | 2 | 3 | 4 |
| have smoked considerably more cigarettes than usual. | 0 | 1 | 2 | 3 | 4 |
| have consumed considerably more drugs (e.g. tranquilizers, sleeping pills or stimulants) than usual. | 0 | 1 | 2 | 3 | 4 |
| have felt a strong desire to consume addictive substances (alcohol, cigarettes, drugs). | 0 | 1 | 2 | 3 | 4 |
| have not been able to control my use of addictive substances (alcohol, cigarettes, drugs). | 0 | 1 | 2 | 3 | 4 |

"Because of the COVID-19 pandemic, **over the past 14 days** I have felt stressed or burdened a lot by…"

|  | Not at all |  |  |  | Very much |
| --- | --- | --- | --- | --- | --- |
| 1. the current pandemic. | 0 | 1 | 2 | 3 | 4 |
| 1. living in a small accommodation. | 0 | 1 | 2 | 3 | 4 |
| 1. being in quarantine. | 0 | 1 | 2 | 3 | 4 |
| 1. childcare. | 0 | 1 | 2 | 3 | 4 |
| 1. taking over school lessons. | 0 | 1 | 2 | 3 | 4 |
| 1. the curfew. | 0 | 1 | 2 | 3 | 4 |
| 1. being in home office. | 0 | 1 | 2 | 3 | 4 |
| 1. customer service. | 0 | 1 | 2 | 3 | 4 |
| 1. worries about my health. | 0 | 1 | 2 | 3 | 4 |
| 1. worries of not being able to get medical care. | 0 | 1 | 2 | 3 | 4 |
| 1. increased conflicts with people close to me. | 0 | 1 | 2 | 3 | 4 |
| 1. financial worries. | 0 | 1 | 2 | 3 | 4 |
| 1. uncertainties regarding my job, training place, studies or school. | 0 | 1 | 2 | 3 | 4 |
| 1. fears of what the future will bring, or that I won't be able to cope with everything. | 0 | 1 | 2 | 3 | 4 |

“**Over the past 14 days** I...”

|  | Not at all |  |  |  | Very much |
| --- | --- | --- | --- | --- | --- |
| have maintained a regular daily routine. | 0 | 1 | 2 | 3 | 4 |
| have planned the day as detailed as possible. | 0 | 1 | 2 | 3 | 4 |
| have integrated sports and exercise into my daily life. | 0 | 1 | 2 | 3 | 4 |
| have maintained my social contacts (telephone, visits or video chats). | 0 | 1 | 2 | 3 | 4 |
| have enjoyed the time together with people close to me. | 0 | 1 | 2 | 3 | 4 |
| have focused on my inner strengths, resources, abilities and talents. | 0 | 1 | 2 | 3 | 4 |
| have changed my attitudes about what is really important to me in life. | 0 | 1 | 2 | 3 | 4 |

**“Over the past 14 days** I...”

|  | Not at all |  |  |  | Very much |
| --- | --- | --- | --- | --- | --- |
| have had the feeling that the political leadership was standing up for me. | 0 | 1 | 2 | 3 | 4 |
| have perceived democracy as an effective form of government. | 0 | 1 | 2 | 3 | 4 |
| have had the feeling that public institutions (e.g. police, judiciary) can be relied upon | 0 | 1 | 2 | 3 | 4 |
| have had the feeling that news and reports on the COVID-19 pandemic are being deliberately withheld. | 0 | 1 | 2 | 3 | 4 |
| have perceived politicians as trustworthy. | 0 | 1 | 2 | 3 | 4 |

“**Over the past 14 days** I...”

|  | Not at all |  |  |  | Very much |
| --- | --- | --- | --- | --- | --- |
| have had the feeling that false reports or untruths about the COVID-19 pandemic are being deliberately disseminated on public broadcasting (e.g. radio and television stations). | 0 | 1 | 2 | 3 | 4 |
| have had the belief that there are alternative or secret explanations for current events. | 0 | 1 | 2 | 3 | 4 |
| have had the belief that there is a relation between what is happening and the production and testing of biological weapons. | 0 | 1 | 2 | 3 | 4 |
| have had the belief that what is happening here is the effect of a struggle or competition between different superpowers. | 0 | 1 | 2 | 3 | 4 |
| have had the belief that this infection serves to deliberately reduce the world population, since there are no longer enough resources for everyone. | 0 | 1 | 2 | 3 | 4 |

“**Over the past 14 days** I have had the feeling that…”

|  | Not at all |  |  |  | Very much |
| --- | --- | --- | --- | --- | --- |
| there is greater solidarity and cohesion in our society. | 0 | 1 | 2 | 3 | 4 |
| I am an integral part of our society or community. | 0 | 1 | 2 | 3 | 4 |
| our nation is growing closer together. | 0 | 1 | 2 | 3 | 4 |
